# Stay on the road: from germ cell specification to gonadal colonization in mammals

**DOI:** 10.1098/rstb.2021.0259

**Published:** 2022-12-05

**Authors:** Bernard A. J. Roelen, Susana M. Chuva de Sousa Lopes

**Affiliations:** ^1^ Anatomy and Physiology, Department Clinical Sciences, Faculty of Veterinary Medicine, Utrecht University, Yalelaan 1, 3584CL Utrecht, The Netherlands; ^2^ Department of Biosciences, Biotechnologies & Biopharmaceutics, University of Bari Aldo Moro, Bari, Italy; ^3^ Department of Anatomy and Embryology, Leiden University Medical Centre, Einthovenweg 20, 2333 ZC Leiden, The Netherlands; ^4^ Ghent-Fertility and Stem Cell Team (G-FAST), Department of Reproductive Medicine, Ghent University Hospital, Corneel Heymanslaan 10, 9000 Ghent, Belgium

**Keywords:** primordial germ cell, specification, migration, mammals, embryo, pluripotency

## Abstract

The founder cells of the gametes are primordial germ cells (PGCs). In mammals, PGCs are specified early during embryonic development, at the boundary between embryonic and extraembryonic tissue, long before their later residences, the gonads, have developed. Despite the differences in form and behaviour when differentiated into oocytes or sperm cells, in the period between specification and gonadal colonization, male and female PGCs are morphologically indistinct and largely regulated by similar mechanisms. Here, we compare different modes and mechanisms that lead to the formation of PGCs, putting in context protocols that are in place to differentiate both human and mouse pluripotent stem cells into PGC-like cells. In addition, we review important aspects of the migration of PGCs to the gonadal ridges, where they undergo further sex-specific differentiation. Defects in migration need to be effectively corrected, as misplaced PGCs can become tumorigenic. Concluding, a combination of *in vivo* studies and the development of adequate innovative *in vitro* models, ensuring both robustness and standardization, are providing us with the tools for a greater understanding of the first steps of gametogenesis and to develop disease models to study the origin of germ cell tumours.

This article is part of the theme issue ‘Extraembryonic tissues: exploring concepts, definitions and functions across the animal kingdom’.

## 1. Mechanisms to separate the germline from the soma

### (a) Germ cell determinants

The most prominent differences between germ cells and somatic cells are the occurrence of meiosis in germ cells, which includes homologous DNA recombination and reduction of the number of chromosomes by half [1], and the capacity of germ cells to form a totipotent cell upon fusion with another germ cell (restoring the diploid state). Germ cells are vital components of genetic diversity and hence evolution; however, since germ cells are the drivers of reproduction, there is not much room for modification or diversification, since this could result in infertility.

The formation of the (precursors of) germ cells can occur by two different mechanisms. The fate of the future germ cells is dictated either by maternally deposited products and organelles (together called germ plasm) already present in the oocyte (preformation mode) or by an inductive process after fertilization (epigenesis mode). The inheritance of germ plasm for germ cell formation has been observed in animals as diverse as nematodes (e.g. *Caenorhabditis elegans*), certain insects (e.g. *Drosophila melanogaster*), frogs (e.g. *Xenopus laevis*) and teleost fish (e.g. *Danio rerio*). The inductive mode of germ cell specification has been observed in other insects (e.g. *Carausius morosus*), salamanders (e.g. *Ambystoma mexicanum*) and mammals (e.g. *Mus musculus*). Interestingly, the preformation of germ cells has arisen convergently multiple times through the animal kingdom and would convey a selective advantage where the inductive mode is thought to be the ancestral animal mechanism [2,3]. It has been suggested that the separation of the germ and somatic lineages early in development by preformation allows for more rapid gene evolution and higher speciation rates [4], while based on sequence analyses this hypothesis is not supported [5]. Alternatively, the timing rather than the mechanism of germ cell specification has been suggested to drive species evolvability, with developmentally early germ cell specification such as observed in rodents allowing high speciation rates [6].

In animals displaying preformation as a mode of germ cell specification, germ plasm in oocytes consists of mitochondria together with maternally deposited RNA-rich membrane-less condensates, called germ granules, processing (P) granules or nuage. These condensates are mainly composed of coding and non-coding RNA and small RNA-associated proteins [7]. The proteins involved are predominantly Tudor domain-containing (TDRD) proteins and P-element-induced wimpy testis-like (PIWIL) proteins. Interestingly, the germ granules behave as liquid-like condensates that can undergo liquid phase separation from the cytoplasm, a process whereby distinct types of molecules can be kept in one place and another set of molecules in another place, resembling oil droplets in a bowl of soup. It has been demonstrated that in *C. elegans*, the P granules that carry information for germ cell formation behave as lipid droplets and can be spatially distributed by dissolution and condensation [8].

In mice, with epigenesis as a mode for germ cell specification, the expression of TDRD and PIWIL is also germline specific [9], and mouse fetal germ cells exhibit different types of P granules [10]. Moreover, it has been suggested that two different types of condensates fuse to form the chromatoid body, a perinuclear structure observed in round spermatids in adult mice. Here, the TDRD7 protein may be a key component and the chromatoid body important for RNA silencing mediated by a specific class of small non-coding RNA, the PIWIL-interacting RNA (piRNA) [11]. In zebrafish, proteins with prion-like domains (PRDs) such as Bucky ball (BUC) together with TDRD proteins are important for phase separation [12]. Concluding, the molecular machinery necessary for epigenesis seem to have retained molecular functionality in animals with the preformation mode of germ cell formation, but is involved in different processes during gametogenesis.

### (b) Lineage specification versus lineage restriction

**Figure 1.**
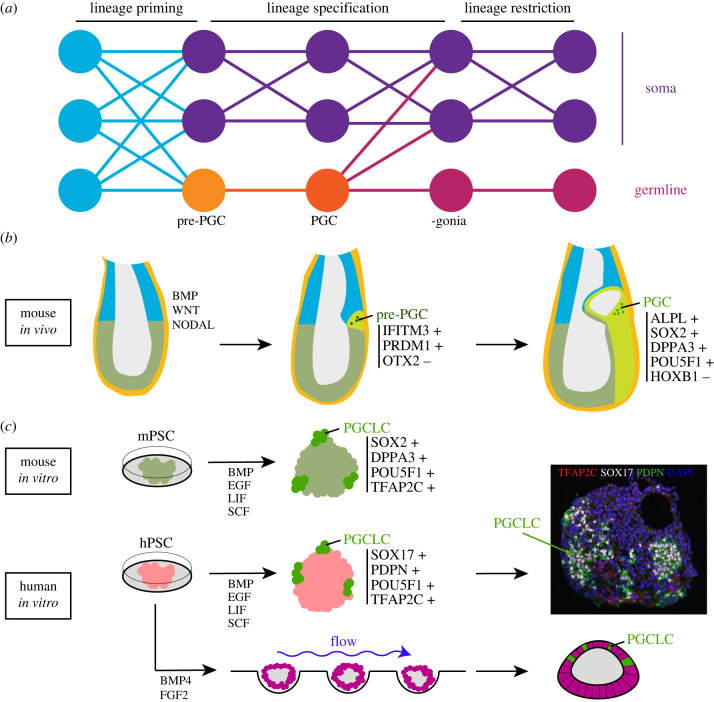
Primordial germ cell (PGC) induction *in vivo* and *in vitro*. (*a*) Schematic representation of the lineage priming, specification and restriction of the germline in mammals. The term ‘-gonia’ refers to oogonia and pre-spermatogonia. (*b*) Events that lead to PGC specification in mouse are initiated by the production of BMP and NODAL in the extraembryonic tissues and WNT in the proximal posterior epiblast just prior to gastrulation. At the beginning of gastrulation pre-PGCs that express IFITM3 and PRDM1, but lack OTX2, are formed and increase in number either by proliferation or further induction, until 7.5 days post-fertilization (dpf), when lineage restriction occurs and a set of specific markers are expressed, such as ALPL, SOX2, DPPA3 and POU5F1, whereas other markers need to be absent, such as HOXB1. (*c*) A robust model of mouse PGC-like cell (PGCLC) specification, using embryoid bodies, is widely used and the mouse PGCLCs have been shown to be able to mature to functional mouse (female and male) gametes. Different models have been developed to investigate the development of human PGCLCs, such as embryoid bodies and one model for amniotic sac development.

There are no agreed definitions regarding germline specification or restriction. Hence, we would like to propose that when considering the germline several events should be distinguished: the priming of embryonic cells to the germline fate (lineage priming), the moment when embryonic cells can no longer contribute to the germline but this commitment can still be reversed (lineage specification), and the time point when germ cells can no longer contribute to somatic cells (lineage restriction or lineage determination) (figure 1*a*). In mammals, lineage specification and lineage restriction may be two distinct events, as has been hypothesized for other animals [13–15].

Most of what is known on the specification of germ cells in mammals comes from studies in the mouse (figure 1*b*), where it was established, using lineage tracing experiments, that primordial germ cells (PGCs) originate from proximal epiblast cells close to the extraembryonic ectoderm [16]. In addition, transplantation studies have demonstrated that prior to gastrulation at least part of the population of more distal epiblast cells also have the capacity to form PGCs, and that the location, close to the inducing extraembryonic cells, is crucial [17,18]. In particular, bone morphogenetic protein (BMP) signalling (from extraembryonic ectoderm and visceral endoderm) is important for PGC formation in mice [19–23]. Simultaneously, restrictive signalling via the anterior visceral endoderm prevents more distal epiblast cells from adapting a PGC fate [24]. In the mouse, the first PGC precursors, referred to as pre-PGCs [25], are thought to be primed to the germline at 6.25 days post-fertilization (dpf) by the upregulation of *Prdm1* (also known as *Blimp1)* expression in response to BMP signals [26]. All the emerging PRDM1-positive cells, between 6.2 and 7.2 dpf, that co-express IFITM3 (also known as FRAGILIS), but do not yet express DPPA3 (also known as STELLA or PGC7) and ALPL (also known as TNAP) could be considered pre-PGCs (figure 1); DPPA3 and ALPL are only upregulated at 7.2 dpf in specified PGCs [24,27]. It is of note that beyond a correlation with the expression of certain marker genes, there are no functional criteria to differentiate pre-PGCs from PGCs, and the two are often referred to as PGCs. We propose that the event of PGC specification determines the end of lineage priming, meaning that no more embryonic cells can enter the germline (figure 1*a*), or in other words no more cells can become ‘blimped’ [28].

Lineage tracing experiments suggested that pre-PGCs, although primed, are not yet lineage restricted and can still give rise to embryonic somatic cells and extraembryonic mesoderm, including the allantois, up until 7.2 dpf [16], none of the clones labelled at 6.5 dpf that contained descendants in PGCs was exclusively formed by PGCs. It seems that PGCs are not lineage restricted in the sense that they can still contribute to other lineages (figure 1*a*). In this regard, it would be interesting to transplant migratory PGCs into younger embryos to investigate the timing of lineage restriction.

Whether PGCs are still able to contribute to somatic lineages in the embryo (meaning that PGCs are not lineage restricted yet) remains to be equivocally established. Interestingly, PGCs express several markers of pluripotency (such as POU5F1 and NANOG), have the potency to develop into teratomas containing different cell lineages and have the ability to reprogram into pluripotent embryonic germ cell lines. However, PGCs from pre- and post-migratory stages do not contribute to chimeras when combined with morula cells or introduced into blastocyst-stage embryos in mice [29]. Finally, it has recently been suggested that mammalian germ cells (from mouse, human and pig) only become lineage restricted after gonadal colonization (after the transition from PGC to gonia), upon the upregulation of *Dazl* expression that restricts developmental potential [30] (figure 1*a*).

### (c) Induction of primordial germ cells

A series of events need to take place to establish the germ cell lineage. Upon the induction by BMPs, a subset of proximal epiblast cells in the mouse embryo start to express IFITM3 [27]. Moreover, fine tuning of BMP signalling activity (via intracellular activity of SMAD1, SMAD5 and SMAD9) together with NODAL signalling activity (via intracellular activity of SMAD2 and SMAD3) in the posterior proximal epiblast results in the upregulation of WNT3 [24,31] and the downregulation of OTX2 expression [32], both events necessary for the induction of pre-PGC fate as well as the size (and location) of the founding population [25] (figure 1*b*). In agreement with this, the deletion of *Otx2* as well as (visceral endoderm-specific) deletion of *Nodal/Smad2* result in the generation of larger numbers of (pre-)PGCs [25,32,33]. Finally, the expression of EOMES and TBXT in the posterior proximal epiblast seems to promote a suitable niche to allow for the efficient specification of the correct number of pre-PGCs [25].

**Figure 2.**
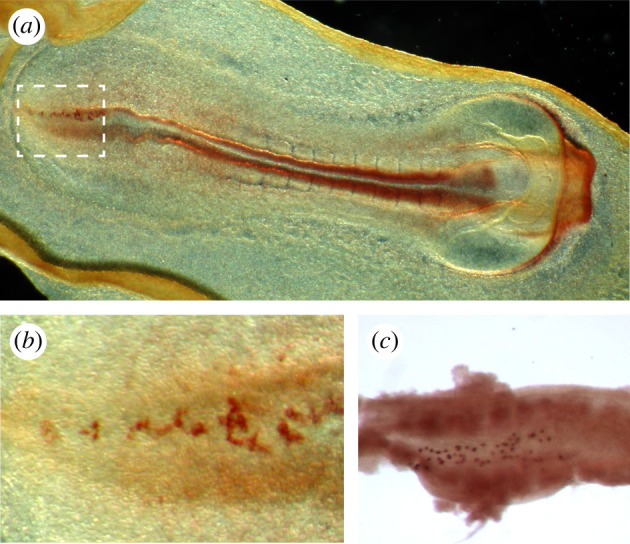
Alkaline phosphatase activity in mammalian embryos. (*a*) Horse embryo 18 dpf stained whole mount for alkaline phosphatase activity, showing staining in the neural tube and PGCs in the posterior part of the embryo (white dashed box). (*b*) Magnification of the white dashed box in (*a*). (*c*) Posterior part of a 9.5 dpf mouse embryo stained whole mount for alkaline phosphatase activity, showing staining in the PGCs in the hindgut.

Within the cluster of IFITM3-expressing cells, a small cluster of about six pre-PGCs that start expressing PRDM1 emerge around 6.25 dpf [26]. Those that start expressing DPPA3 escape from a somatic fate, evidenced by for instance an absence of *Hoxb1* expression [27] (figure 1*b*). Interestingly, DPPA3 is a maternal factor important for the first cleavage divisions, but not essential for PGC formation [34,35]. Other commonly used PGC markers that are also not essential for PGC formation include ALPL [36], IFITM3 [37] and NANOG [38,39]. Moreover, the expression of pluripotency genes *Pou5f1* [40] and *Sox2* [41] are known to be important determinants in (mouse) PGCs. Factors that form a gene regulatory network responsible for PGC specification in mice are *Tfap2c* [42], *Prdm1* [26,43] and *Prdm14* [44]. Importantly, specified PGCs are refractory to BMP signalling occurring in the surrounding extraembryonic mesoderm [25]. In the mouse, the founding population of about 45 pre-PGCs eventually becomes lineage specified at around 7.2 dpf, when the cells reside in the extraembryonic mesoderm at the base of the allantois on top of the posterior part of the primitive streak [16,28]. After specification, PGCs are relatively easy to identify by their alkaline phosphatase activity, caused by the expression of ALPL, which can be visualized by a simple chemical staining procedure in mouse embryos [45], but also in other mammalian embryos (figure 2). Alternatively, mouse PGCs can be identified by the high levels of for instance PRDM1, DPPA3, NANOG or certain surface markers such as SSEA1 and ITGB3 [46]; and human PGCs by for instance SOX17, TFAP2C or certain surface markers such as PDPN, EPCAM and ITGA6 [47–49].

In contrast with mice, the origin of PGCs in human embryos is not entirely clear, but in analogy to the mouse embryo the epiblast has long been considered the tissue of PGC specification. However, the analysis of 11 dpf embryos from the Cynomolgus monkey (*Macaca fascicularis*), a non-human primate, revealed the presence of PGCs in the amniotic ectoderm prior to the onset of gastrulation [50]. Interestingly, in humans and monkeys, the amniotic ectoderm is segregated from the epiblast by cavitation early during development, and the amniotic cavity is immediately sealed [51,52]. The amniotic ectoderm is the third extraembryonic lineage to segregate, after the trophectoderm and the hypoblast, but prior to the formation of extraembryonic mesoderm and the initiation of gastrulation [51,52]. BMP4 is also expressed by the early amniotic ectoderm in monkey and human embryos, indicating that although the location might differ, the inductive signals are homologous in mammals [50]. Interestingly, in pig embryos, the PGCs seem to emerge at the posterior part of the primitive streak [53], more comparable to mouse embryos. Importantly, in pig embryos, the amniotic ectoderm is not formed by cavitation of the epiblast, but similar to mouse (and chicken), the amnion and chorion emerge from the formation of the amniochorionic fold [54–56].

In the cricket *Gryllus bimaculatus,* belonging to the hemimetabolous insect order Orthoptera, inductive signalling is important for PGC formation. Similar to the mouse, the first signals occur via members of the BMP family. The BMP4 orthologues Decapentaplegic (Dpp)1 and Dpp2 and the BMP8B orthologue Glass bottom boat (Gbb) are expressed in the dorsolateral margins of cricket embryos, and knockdown of these factors by embryonic RNAi (eRNAi) led to reduced PGC numbers [57]. While in the mouse it is important to repress *Hoxa1* and *Hoxb1* expression for epiblast cells to develop into PGCs, similarly in crickets the inhibition of *Antennapedia* (*Antp*), *Ultrabithorax* (*Ubx*) and *abdominal-A* (*abd-A*) Hox gene expression led to supernumerary (and ectopic) PGC formation [58]. The expression regulation of these genes is important to assign the PGC-bearing segments and ultimately coordinate the PGC numbers in these segments [58], strongly indicating that somatic cell fate and associated gene regulatory networks that need to be suppressed in the germline to allow proper development, and the molecular mechanisms for doing so, are largely conserved.

### (d) Model systems to study germ cell induction

The use of human embryos for scientific research remains an ethically sensitive issue and those can only be cultured *in vitro* until 14 dpf [59], about the time of PGC specification in humans [52,60]. Hence, this process in humans has been rather challenging to study, also due to the fact that robust models of peri-implantation, whereby the human embryo retains its three-dimensional shape and recognizable morphology, are currently lacking [61,62]. Instead, we have long relied on information from mouse embryos, PGCs and even functional gametes that have been differentiated *in vitro* from mouse pluripotent stem cells (PSCs) [63–65]. The adaptation of protocols to differentiate PGC-like cells (PGCLCs) from PSCs from mouse [63–65] to human has proved successful (figure 1*c*) and has broadened our understanding of the molecular mechanisms that result in PGC/PGCLC induction in humans, even though the efficiency remains low [48,66–68]. As long as the factors needed for induction (BMP4, KITLG, EGF and LIF) were present in the culture medium [66,68], human PSCs differentiated in embryoid bodies gave rise to clusters of PGCLCs (figure 1*c*).

It remains unclear whether *in vivo* human PGCs emerge from the epiblast or the (extraembryonic) amniotic ectoderm, as reported in Cynomolgus monkey [50,51]. Recent growing interest in the development of *in vitro* models to mimic aspects of human early development using hPSCs [69] may shed some light in this issue. Different types of *in vitro* models (hPSCs maintained in different pluripotency states and differentiated under different self-organizing conditions) have emerged to study different periods of human early development. For instance, hPSCs have been used to generate blastoids (proxy of human blastocyst), two-dimensional gastruloids (proxy of the human embryonic disc) and three-dimensional gastruloids (proxy of the elongating embryo) [70]. One of these emerging *in vitro* models, the amniotic sac embryoid model, seems particularly interesting to investigate the formation of the amniotic ectoderm, the initiation of gastrulation and the specification of PGCs [71]. This *in vitro* model uses microfluidics and microwells made of hydrogel to induce the generation of hollow spheres of hPSCs (figure 1*c*). The cells in contact with the hydrogel differentiate into epiblast-like cells and the cells in contact with the medium-flow differentiate into amniotic ectoderm-like cells. Interestingly, although developing (TFAP2C+ NANOG+ SOX17+) PGCLCs were initially observed predominantly in the amniotic ectoderm, those moved to the junction between the amnion and epiblast [71]. Finally, it is pertinent to mention that a unique human embryo at 16–19 dpf has been used for single-cell transcriptomics and has provided an extraordinary dataset to explore the molecular signatures of the different cell types present in the human embryo during gastrulation, including the PGCs [72]. This dataset will be of particular use to understand the similarity of the *in vitro* derived cells with *in vivo* counterparts.

## 2. Germ cell migration

### (a) Finding the way to the gonads

In mouse embryos at 8 dpf, PGCs migrate from their location at the border between the extraembryonic and embryonic tissues, at the base of the allantois, through the developing gut endoderm to the developing gonads. Initial PGC migration seems to be passive, with cells following the morphogenetic movement of the surrounding tissues. Early studies in mice revealed that the expression of the transmembrane tyrosine kinase receptor KIT, coded by the *W* locus, is important for PGC migration and survival [73]. The ligand for KIT, KITLG (SCF or *St* locus in mice), is expressed by the surrounding mesenchyme [74]. Alternative splicing leads to transmembrane and soluble forms of the protein; shortly before and during PGC migration both forms are present in surrounding tissue and are needed for PGC survival [74,75]. The transmembrane KITLG is suggested to provide a niche for migrating PGCs and would maintain their motility essentially by establishing a high local concentration [76]. Similarly, in men increased apoptosis in testes and reduced sperm counts have been associated with the decreased expression of KIT and KITLG [77] and in adolescent varicocele patients, reduced KIT expression has been observed in the tubular compartments of the testes [78], indicating a role in germ cell survival.

**Figure 3.**
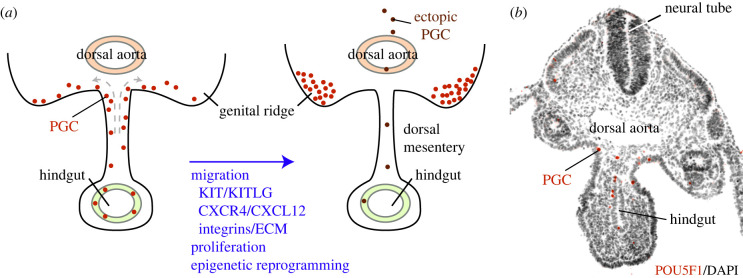
PGC migration. (*a*) PGCs leave the hindgut and migrate through the dorsal mesentery towards the dorsal aorta and round the coelomic angle to colonize the left or right gonadal ridge. During migration, several molecular mechanisms are in place to guide the PGCs, such as the KIT/KITLG, CXCR4/CXCL12 and certain combinations of integrins/ECM. During this period, PGCs proliferate and undergo epigenetic reprogramming (DNA methylation and exchange of histone marks). Some PGCs fail to reach the gonads, remaining ectopically in midline locations and need to activate mechanisms to undergo apoptosis. (*b*) Histology section of human embryo at four–five weeks of development showing migratory POU5F1 + PGCs. ECM, extracellular matrix.

From 9.5 dpf onwards in mouse embryos [79] and around 4–5 weeks of development in humans [80], the PGCs leave the hindgut, move through the dorsal mesentery, emerge into the dorsal body wall and start to colonize the (left and right) genital ridges (figure 3). In order to reach the correct destination, PGCs make use of specific guidance mechanisms. In the mouse, these are provided by the ligand–receptor interaction of CXCL12 and its G protein-coupled receptor CXCR4 [79,81,82]. In developing mouse embryos, *Cxcr4* gene expression has been detected in PGCs from 10.5 dpf onwards. The ligand CXCL12 is predominantly expressed along the dorsal tissues and the mesonephros in mouse embryos. Transverse slices of 9.5 dpf mouse hindgut regions cultured for 20 h demonstrated migration of PGCs via two lateral streams to the genital ridges. When these slices were cultured in the presence of CXCL12, the PGCs emerged from the hindgut but remained scattered along the midline, indicating the guidance function of this protein [82]. Conversely, in mouse embryos homozygous for a targeted mutation of *Cxcr4*, the number of PGCs that reached the genital ridges during development was severely compromised. Since the number of PGCs around the time of migration was also reduced in *Cxcr4-/-* mice, it was concluded that CXCR4 is also needed for PGC proliferation and survival during migration. In agreement, CXCL12 treatment of hindgut slices resulted in increased PGC numbers [82]. Wingless-related MMTV integration site 5a (WNT5A), expressed by somatic cells and binding to receptor tyrosine kinase-like orphan receptor 2 (ROR2) expressed by PGCs, has also been associated with PGC migration. A large proportion of PGCs were retained in the hindgut in *Wnt5a*-defective mouse embryos at 10.5 dpf, while PGCs were also detected in ectopic locations in these animals [83]. In PGCs, WNT5A stimulates migration while simultaneously repressing proliferation, possibly reducing events that compromise migration while associated with cells division, such as loss of adhesion [84].

During migration, PGCs adhere to the extracellular matrix (ECM) [85] and indeed ECM receptors are crucial for germ cell migration. Mouse embryonic cells that lack integrin *Itgb1* can become PGCs, but their migration towards the genital ridges is severely hampered [86]. Interestingly, mouse PGCs that lacked integrin subunits *Itga3*, *Itga6* or *Itgav* migrated normally [86], while both integrin subunits *Itga6* and *Itga6a* are expressed in the developing gonads at 12.5 dpf [87].

It has been suggested that in human embryos, PGCs follow peripheral autonomic nerve fibres during migration from the dorsal mesentery to the gonadal anlagen because of the intimate contact of PGCs with bundles of autonomic nerve fibres [88,89]. Possibly the nerve cells or their Schwann cells produce chemical signals for migration guidance. In a non-human primate, the common marmoset monkey (*Callithrix jacchus*), nerve cells only appear in the vicinity of the gonads after the PGCs have already colonized these. In addition, in embryos of the common marmoset monkey, the distance between PGCs and the closest nerve fibres was at least 50 µm [90]. Similar observations were reported for mouse embryos [90], which would refute the hypothesis that nerve cells act as guiding cues for migrating PGCs, or at least that this system is not evolutionarily conserved.

### (b) Epigenetic reprogramming during migration

One important process that occurs in the PGCs during migration is epigenetic reprogramming. Once specified, the DNA of the PGCs becomes demethylated in a genome-wide manner, except in some specific regions, such as genomic imprinted regions, the silent X chromosome (in female PGCs) and retrotransposon regions [91,92]. The DNA methylation marks on the different genomic imprinted regions (as well as on the silent X chromosome in female PGCs) are erased after the germ cells colonize the gonads and completed by 13.5 dpf, but this process initiates during the migration period [91–93]. A similar pattern of DNA demethylation has been described in other mammalian species, such as pig [94]. The DNA demethylation seems to occur across the entire genome and is more likely to result from active DNA demethylation rather than from replication-dependent passive demethylation [91]. During migration, mouse PGCs also remodel their histone marks. PGCs lose histone 3 lysine 9 trimethylation (H3K9me3) while gaining histone 3 lysine 27 trimethylation (H3K27me3), suggesting a differential role for these two silencing histone marks [93]. When the PGCs arrive at the gonads at 10.5 dpf, they show a pronounced peak of high levels of the active histone marks histone 3 lysine 4 methylation (H3K4me) and histone 3 lysine 9 acetylation (H3K9ac) [93]. Notably, although male and female PGCs are rather similar during this period, female PGCs start reactivating the inactive X chromosome [95,96] as an additional epigenetic process that does not occur in males.

Regarding epigenetic reprogramming in human PGCs, the timing and nature of events that take place during migration are less clear as the availability of human embryos showing migratory PGCs is limited (figure 3*b*), and *in vitro* there are currently no models available to investigate the process of PGC migration in humans. However, when in the gonads, human fetal germ cells (five–nine weeks of development), similar to mouse, seem to have undergone genome-wide DNA demethylation (excluding genomic imprinted region, retrotransposon regions and the silent X chromosome in females) and show high levels of H3K27me3 and H3K4me2 [97–100].

It is interesting to note that there seems to be a level of anti-correlation between epigenetic reprogramming progression and underlying pluripotency characteristics of (mouse) PGCs during migration. Pluripotent embryonic germ cells are most efficiently derived from PGCs isolated from 7.5–8.5 dpf [101], prior to the vast majority of epigenetic reprogramming and indeed migration. By contrast, 10.5–13.5 germ cells are much less efficient at deriving pluripotent embryonic germ cells [101–103], suggesting that the progression of epigenetic reprogramming may in fact contribute to reducing the expression of the pluripotency network in PGCs.

### (c) Stay on the road

Not all migrating PGCs find their way to the gonadal ridges and during their journey a significant number of PGCs are left in the hindgut, dorsal body wall, mesonephros, peri-aortic region, adrenal glands or close to developing gonads in humans [88,104] and mice [105,106] (figure 3*a*). In mice, ectopic PGCs that end up in the adrenal glands can initiate meiosis regardless of the sex [107], but in humans, meiotic entry of ectopic PGCs in the adrenal glands has not been observed [104]. Since these PGCs express many pluripotency genes, such as *POU5F1* and *NANOG*, they can develop into germ cell tumours if they do not receive the correct signals in the gonads to differentiate further [108]. In order to prevent tumour formation of germ cells that have failed to arrive in the genital ridges, a molecular mechanism needs to be in place to ensure that ectopic germ cells are eliminated. Several survival and apoptotic mechanisms have been identified in the mouse. Cyclosporin A has been demonstrated to promote the survival of PGCs by inhibiting the permeability of transition pores in the outer mitochondrial membranes [109]. In addition, fibroblast growth factor signalling suppressed apoptotic cell death, at least *in vitro* [109]. In 10.5 dpf mouse embryos, PGCs in the midline area demonstrated a 3.7-fold increase in the percentage of cells undergoing apoptosis compared with lateral PGCs [110], suggesting that mislocated PGCs are removed by apoptosis. Indeed, in mice with a targeted deletion of *Bax* specifically in PGCs, a significantly increased number of ectopic PGCs were reported [105,106]. Conversely, correctly migrating cells have to be protected from apoptosis and, at least in mice, it is thought that KITLG (in both sexes), NANOS3 (in both sexes) and NANOS2 (in males) are important for this protection during PGC migration [110–112]. Midline PGCs maintain the expression of KIT, the receptor for KITLG, hence PGC survival seems to depend on their localization [110]. Interestingly, by 18.5 dpf most ectopic PGCs have disappeared in the mouse, indicating that the mechanisms that ensure the removal of ectopic PGCs are efficient and in place [105,106]. Even in the absence of BAX, extragonadal germ cells are eliminated and do not lead to extragonadal germ cell tumours, although the mechanisms of removal remain unknown [105].

Neoplasms derived from germ cells can occur in young patients and in adults, both within and outside the gonads. It remains a matter of debate whether the extragonadal germ cell tumours are indeed formed from neoplastic germ cells that were misplaced during migration towards the gonads, metastases from gonadal tumours or, for instance, incompletely differentiated inner cell mass cells [113]. In general, seven types of germ cell tumours can be distinguished, mainly depending on the timing and location of formation [108,114], and it could very well be that these have different origins. It is debatable whether germ cell tumours are formed from abnormal germ cells that acquire pluripotency, or from germ cells with underlying levels of pluripotency that fail to differentiate further. However, in mice, early PGCs give rise to PSCs when cultured *in vitro* [101] with much higher efficiency than spermatogonial stem cells [115]. This would suggest that germ cell tumours arise from (primordial) germ cells that fail to differentiate.

## 3. Conclusion

PGCs, being the precursors of the gametes, are cells of fundamental importance. Although we have greatly broadened our understanding of the germline during the past decade, it is striking how elusive these cells remain, perhaps due to the inaccessibility of the embryo at its early implantation stages, the small size of the embryo and the limited number of PGCs. Moreover, it remains unclear whether the available (and invaluable) *in vitro* models, even in mouse, mirror all the events regarding lineage priming, specification and restriction faithfully; or simply allow the transition to specific germ cell stages, without necessarily passing through all the intermediate steps. Moreover, there is currently a lack of *in vitro* models (in mouse and human) to study PGC migration, and in the future making use of microfluidics platforms or combining hindgut/intestinal organoids with PGCs may result in innovative assays to investigate not only PGC migration, but also pluripotency characteristics and tumorigenic potential during normal and abnormal migration. The period of PGC migration, both in terms of germ cell biology and the communication with the niche remains poorly understood, but investigating mechanisms to guide migration and ensure elimination of ectopic PGCs could provide important cues to the origin of extragonadal germ cell tumours.

## Data Availability

This article has no additional data.
